# Poor-Law Almoners

**Published:** 1920-07-24

**Authors:** 


### Poor-Law Almoners.

APPOINTMENT AT LEWISHAM.

THE Lewisham Board of Guardians has now decided that the Medical Superintendent may admit cases without an order from the relieving officer on the production of a certificate from a private practitioner, and that in urgent cases this certificate shall not be necessary. To make the infirmary still more like a hospital, the collector, whose duty is to inquire into the capacity of patients to contribute to the cost of their treatment, is to be called the almoner of the hospital.

The cases which the district medical officers attend will still be admitted on the order of the relieving officer.

